# Is computer-assisted total knee replacement for beginners or experts? Prospective study among three groups of patients treated by surgeons with different levels of experience

**DOI:** 10.1007/s10195-012-0205-z

**Published:** 2012-07-18

**Authors:** Norberto Confalonieri, Cesare Chemello, Pietro Cerveri, Alfonso Manzotti

**Affiliations:** 1 Ist Orthopedic Department, C.T.O. Hospital, Via Bignami 1, 20100 Milan, Italy; 2 Azienda Ospedaliera di Padova Clinica Ortopedica, Via Giustiniani 2, 35123 Padua, Italy; 3 Bioengineering Department, Politecnico di Milano, P.zza Leonardo da Vinci, 20100 Milan, Italy; 4 Via G. Berchet, 9, 35131 Padua, Italy

**Keywords:** Navigation future, Total knee replacement, Learning curve, Black box, Cutting errors, Computer assistance

## Abstract

**Background:**

Computer-assisted total knee replacement (TKR) has been shown to improve radiographic alignment and therefore the clinical outcome. Outliers with greater than 3° of varus or valgus malalignment in TKR can suffer higher failure rates. The aim of this study was to determine the impact of experience with both computer navigation and knee replacement surgery on the frequency of errors in intraoperative bone cuts and implant alignment, as well as the actual learning curve.

**Materials and methods:**

Three homogeneous groups who underwent computer-assisted TKR were included in the study: group A [surgery performed by a surgeon experienced in both TKR and computer-assisted surgery (CAS)], B [surgery performed by a surgeon experienced in TKR but not CAS], and C [surgery performed by a general orthopedic surgeon]. In other words, all of the surgeons had different levels of experience in TKR and CAS, and each group was treated by only one of the surgeons. Cutting errors, number of re-cuts, complications, and mean surgical times were recorded. Frontal femoral component angle, frontal tibial component angle, hip–knee–ankle angle, and component slopes were evaluated.

**Results:**

The number of cutting errors varied significantly: the lowest number was recorded for TKR performed by the surgeon with experience in CAS. Superior results were achieved in relation to final mechanical axis alignment by the surgeon experienced in CAS compared to the other surgeons. However, the total number of outliers showed no statistically significant difference among the three surgeons. After 11 cases, there were no differences in the number of re-cuts between groups A and C, and after 9 cases there were no differences in surgical time between groups A and B.

**Conclusion:**

A beginner can reproduce the results of an expert TKR surgeon by means of navigation (i.e., CAS) after a learning curve of 16 cases; this represents the break-even point after which no statistically significant difference is observed between the expert surgeon and the beginner utilizing CAS.

## Introduction

Nowadays, to obtain the best positioning of the components during a total knee replacement (TKR), the surgeon must use the best technology. Malalignment can adversely effect the longevity of knee prostheses, causing early wear and implant loosening, both of which are linked to suboptimal implant positioning [1–3]. Greater than 3° of varus or valgus malalignment in total knee replacement can result in higher failure rates, whilst correct alignment has been associated with improved clinical outcome [3–5]. Several authors have shown that traditional hand-guided alignment systems can produce potential errors in the bone-cutting process, even when used by experienced surgeons [6–11]. The use of a navigation system could help the young surgeon and the expert surgeon to achieve good, long-lasting results.

Recently, Manley et al. [12] showed that patients undergoing TKR in low-volume hospitals (1–25 procedures/year) had a higher risk of early revision at five and eight years compared with those performed in hospitals with the highest volumes (>200 procedures/year). Total knee replacement performed with computer-aided alignment appears to produce superior radiological results to hand-guided techniques [13–15]. These computer-assisted surgery (CAS) systems have been shown to both improve mechanical alignment and reduce outliers. Both of these outcomes are linked to a potential decrease in the TKR revision rate. Computer navigation provides continuous feedback during all phases of knee replacement surgery, providing an opportunity to correct any bone-cutting errors. Using a navigation system implies the application of an authentic protocol. There are obligatory steps to be carried out when using the computer; every surgeon must perform the same steps, and every step is recorded, so we can check what has been performed with precision. It has been suggested that computer navigation could be used as a teaching tool, so that even an inexperienced TKR surgeon would be able to perform more expertly [6, 16, 17]. In 2008, in a retrospective study, Yau et al. failed to show any improvement in postoperative alignment using a computer-assisted technique in a low-volume knee practice [18]. In 2005, Daubresse et al. hypothesized that the learning curve for a computer-navigated TKR technique cannot be longer than that of the free-hand technique, even in a community hospital [19].

The aim of this study was to determine the break-even point for different experiences in TKR and CAS. The frequency of intraoperative bone cut errors, the final implant alignment, and the surgical time were assessed to evaluate the learning curve.

## Materials and methods

A prospective study of 75 selected patients undergoing computer-assisted TKR was undertaken. Strict inclusion criteria were adopted in the study; only patients with primary osteoarthritis, a body mass index of ≤35, a maximum mechanical axis deformity of less than 15°, and at least 90° of knee flexion were included. Before the study we assigned each patient into one of three equally sized groups (groups A, B, and C; see Table 1). Each patient was informed and gave consent prior to being included in the study. The study was authorized by the local ethical committee and was performed in accordance with the ethical standards of the 1964 Declaration of Helsinki as revised in 2000.

Group A had their surgery performed by a surgeon experienced in both knee replacement (more than 70 TKRs/year) and computer-navigated (more than 250 CAS-TKRs implanted) surgery. The surgeon for group B patients was experienced in knee replacement (more than 70 TKRs/year) but had not previously performed computer-guided surgery. A general orthopedic surgeon performed all TKRs in group C. This surgeon had a low-volume TKR experience (less than 20 TKRs/year) and no previous experience with computer-guided surgery. The same computer navigation system (Vector Vision, version 1.52, BrainLAB, Munich, Germany) was used in all TKRs. The prosthesis used in all patients was the same (Genesis II, Smith and Nephew, Memphis, TN, USA). Each surgeon involved in the trial was never involved in the operations of the other two.

**Table 1:** Patient characteristics

	Group A	Group B	Group C	*p*
Body mass index	31.36 SD ±2.78 Range 26–35	31.72 SD ±2.44 Range 26–35	31.20 SD ±2.86 Range 26–35	0.79
Preoperative flexion (°)	105.4 SD ±9.67 Range 90–120	109.8 SD ±10.25 Range 90–120	107.6 SD ±8.43 Range 95–120	0.23
Preoperative HKA angle (°)	170.84 SD ±5.04 Range 165–183	171.96 SD ±5.26 Range 172–180	174.4 SD ±4.79 Range 166–183	0.38

A standardized operative approach was followed in all TKRs. In all cases, the midline patellar skin incision was pre-drawn to a length ranging from between 13.5 and 15.5 cm. A medial para-patellar arthrotomy was extended proximally to the quadriceps tendon in all patients. The patellar was retracted laterally in each case. All prostheses were implanted using the same dedicated instruments, including cutting blocks specifically designed for computer navigation. The cutting blocks were fixed with a combination of treated and smooth pins in all patients. All implants were cemented. No patients underwent patellar resurfacing. The same pre- and postoperative rehabilitation protocols were used in all three groups. Early weight-bearing was encouraged in all patients if tolerated.

**Fig. 1:**
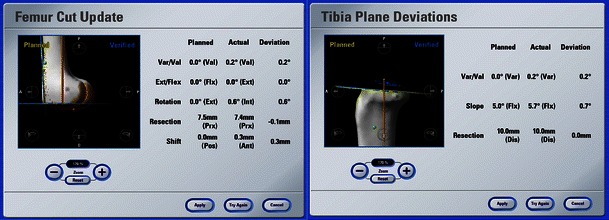
Intraoperative control of the cuts

For each implant, the tibial and femoral cutting errors and the number of re-cuts were recorded. A cutting error occurred when the planned angle of the bone cut as measured by the cutting block differed from the angle seen after sawing. The cutting error was measured in the frontal and sagittal planes of both the tibia and femur, giving four measurements (Fig. 1). According to a pre-determined surgical protocol, a re-cut was required if the cutting error was ≥3°. The number of complications and the mean surgical time (time between skin incision and tourniquet release) were measured for each group.

Standing radiographs were performed six months postoperatively with the knee in maximum extension, the patella pointing forward, and both hips and ankles visible on the film. The lateral radiographs were taken with the knee in 30° of flexion on a radiographic film. The radiographs were taken according to a standardized protocol and magnification. If malrotation was detected the radiographs were repeated. An independent radiologist assessed all radiographs.

**Fig. 2:**
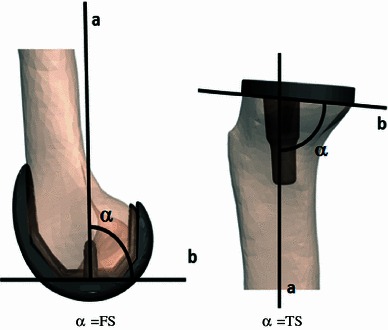
Femoral slope and tibial slope

**Fig. 3:**
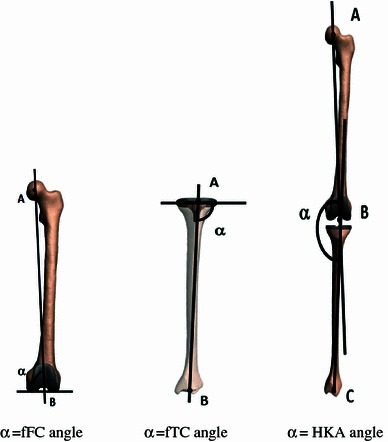
Mechanical axis

The frontal femoral component (fFC) angle, the frontal tibial component (fTC) angle, the hip–knee–ankle (HKA) angle, and the sagittal orientations of components (slopes) were all measured. These parameters were utilized to evaluate the quality of the surgical outcome. The fFC is the angle between the mechanical axis of the femur and the transverse axis of the femoral component. The fTC is the angle between the mechanical axis of the tibia and the transverse axis of the tibial component. The slopes of the femoral (FS) and tibial (TS) components were defined as the angle between a line drawn tangential to the base plate (surface in contact with bone) of the respective component and the anterior femoral cortex or tibial mechanical axis, respectively (Figs. 2, 3). The desired prosthesis alignment for each parameter was determined prior to the study as an fFC angle of 90°, fTC angle of 90°, HKA angle of 180°, femoral slope of 90°, and tibial slope of 87°. The total number of outliers for all five radiological parameters were determined for each group and compared. An outlier was defined as a postoperative malalignment of any parameter of greater than 3° from the target value (Table 2).

**Table 2:** Difference between the desired prosthesis alignment and alignment after first cut; number of re-cuts needed to obtain the correct angle

	Group A	Group B	Group C
Frontal femoral component angle [fFC angle (°)]	1.04 SD ±0.89 Range 0–3 # Re-cuts 2	1.20 SD ±0.87 Range 0–3 # Re-cuts 2	1.32 SD ±0.95 Range 0–3 # Re-cuts 3
Femoral slope [FS angle (°)]	0.52 SD ±0.77 Range 0–3 # Re-cuts 1	0.76 SD ±0.78 Range 0–3 # Re-cuts 1	1.04 SD ±0.98 Range 0–3 # Re-cuts 2
Frontal tibial component angle [fTC angle (°)]	0.80 SD ±0.91 Range 0–3 # Re-cuts 1	0.96 SD ±1.31 Range 0–5 # Re-cuts 4	1.28 SD ±1.28 Range 0–4 # Re-cuts 5
Tibial slope [TS angle (°)]	0.72 SD ±0.79 Range 0–2 # Re-cuts 0	0.88 SD ±0.83 Range 0–3 # Re-cuts 1	1.08 SD ±1.04 Range 0–3 # Re-cuts 3

Statistical analysis of the results was performed and the three groups were compared. Because of an abnormal data distribution, nonparametric testing (Kruskal–Wallis test) was performed using Statistica 7.0 software (StatSoft Inc., Tulsa, OK, USA) for analysis. Statistical significance was set at *p* ≤ 0.05.

## Results

Analysis of the demographic data for all three groups showed no statistically significant differences in preoperative flexion, body mass index, or preoperative deformity (Table 1).

There were no complications relating to the surgical technique or the surgeon’s experience.

Statistically significant differences were, however, seen when the following parameters were compared among groups: distal femoral cut, proximal femoral cut, femoral component slope, mechanical axis. Statistically significantly inferior results were seen for the patients operated on by the general orthopedic surgeon concerning the distal femoral cut in the sagittal plane, compared to the other two groups (*p* = 0.05). No significant difference was seen for the distal femoral cut in the coronal plane among groups A, B, and C. For the proximal tibial cut in the coronal plane, standard deviations of 0.91°, 1.31°, and 1.28° were noted for groups A, B, and C, respectively. These differences were not statistically significant. A statistically significant difference (*p* = 0.007) was seen in the proximal tibial cut in the sagittal plane between the patients operated on by the surgeon experienced in computer-guided and knee replacement surgery and the general orthopedic surgeon (Table 2).

A statistically significant difference (*p* = 0.05) was seen in the femoral component slope between the patients operated on by the experienced TKR surgeons and the general orthopedic surgeon. The slope of the femoral component was 90.36° (range 87–94°), 89.92° (range 88–95°), and 90.68° (range 88–94°) in groups A, B, and C, respectively. There was no significant statistical difference in the postoperative fFC and fTC angles across the three patient groups.

**Fig. 4:**
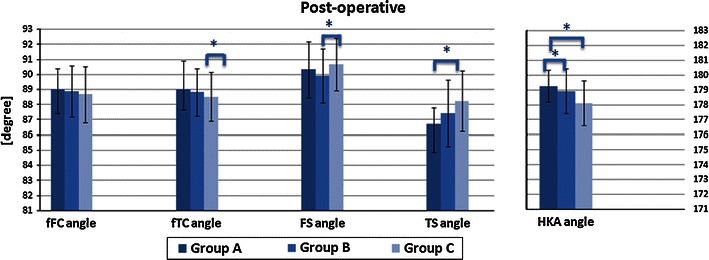
Analysis of postoperative angles

The slope of the tibial component in group A was 86.72° (range 84–91°), in group B it was 87.44° (range 84–92°), and in group C it was 88.24° (range 84–91°). A statistically significant difference (*p* = 0.007) was noted between groups A and C. The patients who underwent TKR performed by the surgeon experienced in both computer-guided and knee replacement surgery had a statistically significantly improved mechanical axis when compared to the patients from groups B (*p* = 0.030) and C (*p* = 0.0006) (Table 3; Fig. 4). Despite these findings, no statistically significant difference was seen among the three patient groups in terms of the total number of outliers for all five radiographic parameters.

**Table 3:** Average postoperative angles

	Group A	Group B	Group C
Frontal femoral component angle [fFC angle (°)]	89.04 SD ±1.62 Range 86–92	88.88 SD ±1.69 Range 86–93	88.68 SD ±1.88 Range 86–93
Frontal tibial component angle [fTC angle (°)]	89.04 SD ±1.37 Range 86–91	88.82 ± 1.59 Range 85–91	88.52 SD ±1.63 Range 85–91
Femoral slope [FS angle (°)]	90.36 SD ±1.89 Range 87–94	89.92 SD ±1.78 Range 88–95	90.68 SD ±1.75 Range 88–94
Tibial slope [TS angle (°)]	86.72 SD ±1.84 Range 84–91	87.44 SD ±2.18 Range 84–92	88.24 SD ±2.00 Range 84–91
Hip–knee–ankle angle [HKA angle (°)]	179.28 SD ±1.06 Range 177–181	178.94 SD ±1.50 Range 177–182	178.12 SD ±1.50 Range 176–183

**Fig. 5:**
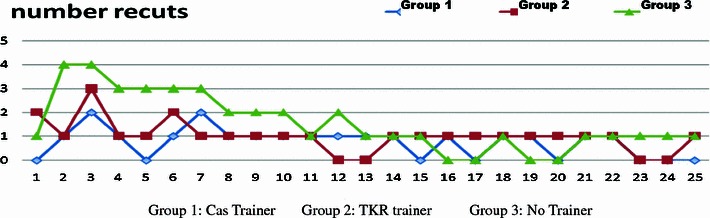
Number of cases after which there was no statistical difference among the surgeons in terms of the number of re-cuts needed (i.e., the break-even point). After 11 cases, the trainee obtained the same results as the expert

There was a correlation between the level of experience in both computer navigation and knee replacement surgery and the number of re-cuts. Four re-cuts were seen in group A, eight re-cuts were needed in group B, and 13 re-cuts were done in group C. A statistically significant difference was seen between groups A and C (*p* = 0.02). This difference suggested an inverse relationship between the surgeon’s experience and the number of re-cuts. We found no statistical difference between the group operated on by the CAS-trained surgeon and the group operated on by the TKR-trained surgeon, and the break-even point between the group operated on by the CAS-trained surgeon and the group operated on by the general orthopedic surgeon corresponded to 11 cases (Fig. 5).

A statistically significant increase in surgical time was seen for the patients in groups B and C (who had TKR performed by surgeons lacking experience in computer-assisted techniques) compared to group A. We observed no statistical difference among the surgeons after nine cases between group A and group B, and after 16 cases between group A and group C.

Summarizing, in group A, we observed statistically significantly superior results regarding the distal femoral cut, the proximal tibial cut, the mechanical axis, the number of re-cuts, and the surgical time when compared with group C; we also noted statistically significantly superior results concerning the mechanical axis and surgical time for group A compared to group B. We saw a statistically significantly improved mechanical axis in group B compared to group C.

No complications were seen in any of the three groups.

## Discussion

Malalignment of a TKR has been shown to adversely influence implant survival. Different intraoperative pitfalls can affect the final postoperative alignment in TKR. Malalignment in the sagittal plane in excess of 3° can increase the implant failure rate and result in poorer clinical outcomes [3, 12]. Using traditional intramedullary alignment systems, deviations of up to 8° can occur in the femoral axis, depending on the size and length of the intramedullary guide [20]. In 2001, Mahaluxmivala et al. showed that TKR alignment improves with surgical experience [8]. Unstable cutting blocks and saw deviations during osteotomy have been shown to result in cutting errors [6, 10]. A strict correlation has been demonstrated between surgical experience of TKR and implant survival [3, 8, 12].

Computer-assisted surgery provides the surgeon with continuous intraoperative feedback on cutting errors and implant alignment during all phases of TKR [6]. Recent studies have demonstrated that computer navigation may play a role in reducing the learning curve in joint replacement surgery [17, 21].

The aim of the current prospective controlled trial was to assess the influence of computer navigation simultaneously on the learning curve, the frequency of intraoperative cutting errors, and component alignment in TKR. The strong points of this study include the use of a standardized surgical protocol in a single orthopedic department and the application of strict inclusion criteria. Obese patients and those with a major preoperative knee deformity were excluded. As such, it is the first study reported in the literature in which an attempt was made to reduce the influence of patient variables on the final result by minimizing these differences preoperatively. A potential weakness of the trial was that the series magnitude was not confirmed by a preliminary power study.

Using a computer navigation system reduces the influence of cutting block stability and saw blade movement on the final result. A reduction in the number of cutting errors has been shown to occur when a navigation system is used for TKR surgery [10]. In agreement with the previous study, we have shown that experience with computer navigation in TKR results in a lower number of intraoperative cutting errors. The number of re-cuts required was greater in the two groups operated on by surgeons with no prior experience in computer-assisted TKR. A statistically significant increase in the number of re-cuts was seen for TKRs performed by the general orthopedic surgeon compared with the surgeon experienced in computer-guided surgery, but we did not find any statistical difference among the group operated on by the CAS-trained surgeon and the other two groups after 11 cases.

Superior alignment and clinical results have been achieved using computer-guided TKR when compared to traditional techniques, even in experienced hands [17, 21–23]. The advantages of computer-guided TKR have not been as clearly demonstrated in low-volume surgical centers. In a retrospective study, Yau et al. [18] did not find any improvement in postoperative TKR alignment with the use of a navigation system in a low-volume practice. The authors stated that the severity of the preoperative deformity affected overall alignment postoperatively. Slover et al. [24] used a Markov decision model to demonstrate that computer navigation is less likely to be a cost-effective investment in healthcare improvement in centers with a low volume of joint replacements.

In our study, the postoperative mechanical axis of the knee was significantly better when the surgeon experienced in computer-assisted TKR performed the surgery. Other postoperative radiological parameters in the coronal plane were similar in all three groups. The accuracy of the tibial and femoral slope cut was affected by the experience of the surgeon. A statistically significantly inferior result was obtained for both of these parameters when the TKR was performed by the general orthopedic surgeon in this study. A possible explanation for this difference, based on the authors’ previous experience with computer-assisted TKR, is that saw inclination is not completely controlled by the cutting block in the sagittal plane. As a result, experience of knee replacement surgery may play an extremely important role in determining the tibial and femoral slopes in particular. Despite this, the overall postoperative TKR alignment was similar for all three surgeons. Each surgeon had a similar number of total outliers, with no statistically significant difference in the number of patients with malalignment exceeding 3° of the target value.

 In 2008, Sampath et al., using a computer-assisted TKR, reported that tourniquet time increased with larger preoperative deformities and a high body mass index, and decreased with surgical experience [21]. Previous studies [12, 17, 18, 21] have shown a significant difference in surgical time, measured between skin incision and tourniquet release, when comparing inexperienced surgeons with those familiar with computer navigation. The current study also demonstrated that surgical time decreased significantly with experience in navigation, but that the intraoperative complication rate did not change.

**Fig. 6:**
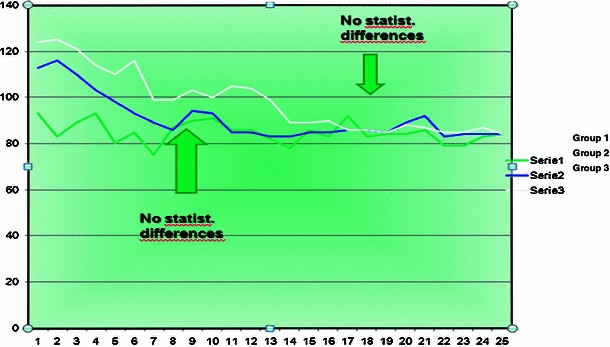
Number of cases after which there was no statistical difference in surgical time (i.e., the break-even point). After 9 cases, the trainee obtained the same results as the expert, and after 16 cases the beginner had caught up with the expert

This study shows that the learning curve needed to perform a TKR with a navigation system is 9 cases for a TKR-trained surgeon and 16 cases for a surgeon who is untrained in both CAS and TKR (Fig. 6).

The authors demonstrated in this study that TKR performed with computer navigation yielded similar postoperative results in terms of overall alignment, even when there were variations in surgical experience. The best recovery of the mechanical axis was achieved when the surgery was performed by a surgeon experienced in computer-assisted TKR. Experience in knee replacement surgery in general leads to statistically superior tibial and femoral slopes when computer navigation is used. Experience with computer-assisted alignment techniques reduces surgical time.

In conclusion, computer navigation appears to be a useful tool in knee replacement surgery, independent of surgical experience, as surgeons with different levels of experience produced the same number of outliers. This study shows that a beginner in TKR can reproduce the correct alignment of a total knee arthroplasty just like an expert TKR surgeon (i.e., there is no statistical difference in the results achieved by the beginner and the expert surgeon) after operating on 11 cases by means of computer navigation. Initially, the surgical time is obviously longer for surgeons with little experience of CAS, but the break-even point corresponded to 16 cases. Therefore, the learning curve achieved with CAS is not as long as the traditional learning curve (Figs. 5, 6).

Technology now allows us to minimize human error in all areas, above all in complex systems. In our experience, even in computer-navigated joint replacement surgery, presenting precise numbers for angles, axes, and spaces can help the surgeon to standardize the surgical procedure. Furthermore, the surgeon must always perform the same surgical steps and use the same controls, like a check list. Every step is recorded, so we have an authentic black box that can show whether a bad result is a genuine human error. CAS is also a very important instructor for young surgeons; they can review every step after surgery, thus gaining an understanding of their mistakes throughout their training.

Longer follow-up will be needed to determine whether better postoperative alignment results in superior clinical outcomes and compensates for higher costs and longer surgical times.

## References

[1] Lewold S, Knutson K, Lidgren L (1993). Reduced failure rate in knee prosthetic surgery with improved implantation technique. Clin Orthop Relat Res.

[2] Lotke PA, Ecker ML (1977). Influence of positioning of prosthesis in total knee replacement. J Bone Joint Surg Am.

[3] Ritter MA, Faris PM, Keating EM, Meding JB (1994) Postoperative alignment of total knee replacement. Its effect on survival. Clin Orthop Relat Res 299:153–156 8119010

[4] Ek ET, Dowsey MM, Tse LF, Riazi A, Love BR, Stoney JD, Choong PF (2008). Comparison of functional and radiological outcomes after computer-assisted versus conventional total knee arthroplasty: a matched-control retrospective study. J Orthop Surg (Hong Kong).

[5] Longstaff LM, Sloan K, Stamp N, Scaddan M, Beaver R (2009) Good alignment after total knee arthroplasty leads to faster rehabilitation and better function. J Arthroplasty 24(4):570–578 10.1016/j.arth.2008.03.002 18534396

[6] Bathis H, Perlick L, Tingart M, Perlick C, Luring C, Grifka J (2005). Intraoperative cutting errors in total knee arthroplasty. Arch Orthop Trauma Surg.

[7] Carter RE, Rush PF, Smid JA, Smith WL (2008). Experience with computer-assisted navigation for total knee arthroplasty in a community setting. J Arthroplasty.

[8] Mahaluxmivala J, Bankes MJ, Nicolai P, Aldam CH, Allen PW (2001). The effect of surgeon experience on component positioning in 673 press fit condylar posterior cruciate-sacrificing total knee arthroplasties. J Arthroplasty.

[9] Otani T, Whiteside LA, White SE (1993). Cutting errors in preparation of femoral components in total knee arthroplasty. J Arthroplasty.

[10] Plaskos C, Hodgson AJ, Inkpen K, McGraw RW (2002). Bone cutting errors in total knee arthroplasty. J Arthroplasty.

[11] Santini AJ, Raut V (2008) Ten-year survival analysis of the PFC total knee arthroplasty—a surgeon’s first 99 replacements. Int Orthop 32(4):459–465 10.1007/s00264-007-0351-8 PMC2532265 17364178

[12] Manley M, Ong K, Lau E, Kurtz SM (2009) Total knee arthroplasty survivorship in the United States medicare population effect of hospital and surgeon procedure volume. J Arthroplasty 24(7):1061–1067 10.1016/j.arth.2008.06.011 18977638

[13] Chin PL, Yang KY, Yeo SJ, Lo NN (2005). Randomized control trial comparing radiographic total knee arthroplasty implant placement using computer navigation versus conventional technique. J Arthroplasty.

[14] Confalonieri N, Manzotti A, Pullen C, Ragone V (2005). Computer-assisted technique versus intramedullary and extramedullary alignment systems in total knee replacement: a radiological comparison. Acta Orthop Belg.

[15] Sparmann M, Wolke B, Czupalla H, Banzer D, Zink A (2003). Positioning of total knee arthroplasty with and without navigation support. A prospective, randomized study. J Bone Joint Surg Br.

[16] Cobb JP, Kannan V, Brust K, Thevendran G (2007). Navigation reduces the learning curve in resurfacing total hip arthroplasty. Clin Orthop Relat Res.

[17] Jenny JY, Mielke RK, Giurea A (2008). Learning curve in navigated total knee replacement: a multi-centre study comparing experienced and beginner centers. Knee.

[18] Yau WP, Chiu KY, Zuo JL, Tang WM, Ng TP (2008). Computer navigation did not improve alignment in a lower-volume total knee practice. Clin Orthop Relat Res.

[19] Daubresse F, Vajeu C, Loquet R (2005). Total knee arthroplasty with conventional or navigated technique: comparison of the learning curves in a community hospital. Acta Orthop Belg.

[20] Reed SC, Gollish J (1997). The accuracy of femoral intramedullary guides in total knee arthroplasty. J Arthroplasty.

[21] Sampath SA, Voon SH, Sangster M, Davies H (2009) The statistical relationship between varus deformity, surgeon’s experience, BMI and tourniquet time for computer assisted total knee replacements. Knee 16(2):121–124 10.1016/j.knee.2008.09.008 19013071

[22] Lüring C, Oczipka F, Perlick L, Tingart M, Grifka J, Bäthis H (2009) Two year follow-up comparing computer assisted versus freehand TKR on joint stability, muscular function and patients satisfaction. Knee Surg Sports Traumatol Arthrosc 17(3):228–232 10.1007/s00167-008-0644-5 18941737

[23] Seon JK, Park SJ, Lee KB, Li G, Kozanek M, Song EK (2009) Functional comparison of total knee arthroplasty performed with and without a navigation system. Int Orthop 33(4):987–990 10.1007/s00264-008-0594-z PMC2898982 18587573

[24] Slover JD, Tosteson AN, Bozic KJ, Rubash HE, Malchau H (2008). Impact of hospital volume on the economic value of computer navigation for total knee replacement. J Bone Joint Surg Am.

